# Downregulation of selective microRNAs in trigeminal ganglion neurons following inflammatory muscle pain

**DOI:** 10.1186/1744-8069-3-15

**Published:** 2007-06-08

**Authors:** Guang Bai, Rajini Ambalavanar, Dong Wei, Dean Dessem

**Affiliations:** 1 Department of Biomedical Sciences, Program in Neuroscience, University Maryland Dental School, Baltimore, MD, USA

## Abstract

Active regulation of gene expression in the nervous system plays an important role in the development and/or maintenance of inflammatory pain. MicroRNA (miRNA) negatively regulates gene expression via posttranscriptional or transcriptional inhibition of specific genes. To explore the possible involvement of miRNA in gene regulation during inflammatory pain, we injected complete Freund's adjuvant (CFA) unilaterally into the rat masseter muscle and quantified changes in neuron-specific mature miRNAs in the trigeminal ganglion (TG). Real-time reverse-transcription polymerase chain reaction revealed significant, but differential, downregulation of mature miR-10a, -29a, -98, -99a, -124a, -134, and -183 in the ipsilateral mandibular division (V3) of the TG within 4 hr after CFA. In contrast, levels of tested miRNAs did not change significantly in the contralateral V3 or the ipsilateral ophthalmic and maxillary divisions of the TG from inflamed rats, nor in the ipsilateral V3 of saline-injected animals. The downregulated miRNAs recovered differentially to a level equal to or higher than that in naive animals. Full recovery time varied with miRNA species but was at least 4 days. Expression and downregulation of some miRNAs were further confirmed by *in situ *hybridization of TG neurons that innervate the inflamed muscle. Although neurons of all sizes expressed these miRNAs, their signals varied between neurons. Our results indicate that miRNA species specific to neurons are quickly regulated following inflammatory muscle pain.

## Background

Inflammation associated with some pathologies may develop allodynia or hyperalgesia defined as an over-reaction to non-noxious or noxious stimuli, respectively [1,2]. Gene expression is an important molecular mechanism underlying inflammatory pain since the measured steady-state levels of mRNA and/or protein in pain/nociceptive pathway in animal models are actively altered during the development and maintenance of pain [2-6]. Our understanding of how individual genes are selectively regulated during inflammatory pain is limited mostly to the regulation of transcriptional control [2]. MicroRNA (miRNA) represents a group of small noncoding RNAs in 18~23 nucleotide sequences. These evolutionarily conserved molecules mainly interfere with gene expression at posttranscriptional levels and moderately promote RNA degradation by acting on specific sequences in the 3' untranslated region of target mRNA, while some of them inhibit gene transcription by participating in chromatin remodeling [7-9]. While many miRNAs have been detected in the nervous system [10-12], their functional significance has been restricted mostly to events involving nervous system development [10,13-18]. Although miRNAs are present in mature neurons, their functionality and regulation remain largely unexplored.

## Findings

To explore the mechanism(s) underlying the gene alteration during inflammatory pain and to investigate the function of miRNA in the adult nervous system, we quantified several neuronal miRNAs in a model of inflammatory muscle pain. We injected CFA (150 μl, oil:saline = 1:1, Sigma, St. Louis, MO) unilaterally into the masseter muscle of male rats (Sprague-Dawley, ~250 gr, Harlan, Indianapolis, IN). This injection produced significant mechanical allodynia while intramuscular injection of saline did not [4,6]. After the development of allodynia, we dissected the ophthalmic and maxillary divisions (V1/2) and V3 from individual TGs. Tissues were combined from two animals and cellular RNA was extracted for miRNA quantification [4]. This design is based on the hypothesis that sensory neurons are a critical component in pain/nociception pathway [1] and sensory neurons innervating mandibular muscle have their perikarya located in V3 [19]. To quantify miRNA, we employed a newly developed TaqMan real-time reverse-transcription polymerase chain reaction (RT-PCR) assay (ABI, Foster City, CA). This technology allows us to specifically measure selective mature miRNAs from nanogram amounts of cellular RNA, thus making it possible to study small tissues such as dissected TG [20]. In the present study we used the following criteria to limit miRNA number from more than 400 identified molecules [21]: First, miRNAs expressed in TG were included. Seven miRNAs were reported previously from TG [11]. Second, those involved in neuronal plasticity [15], one of cellular mechanisms underlying inflammatory pain [22], were chosen. Third, one member per miRNA family was examined [11,12,23]. Fourth, the amount of extracted RNA and the availability of relevant TaqMan miRNA assays limited the number of miRNA tested. Last, they are conserved among human and rodents. In preliminary studies, we examined ten miRNAs from a pool of RNA extracted from TG V3 (n = 16). We were able to detect miR-10a, -29a, -98, -99a, -124a, -134, and -183, but not miR-122, miR-143, and miR-153 even after 50 cycles of PCR, although they were previously reported from TG via Northern analysis [11]. In parallel experiments, all assays produced robust signal from brain RNA (data not shown).

**Figure 1:**
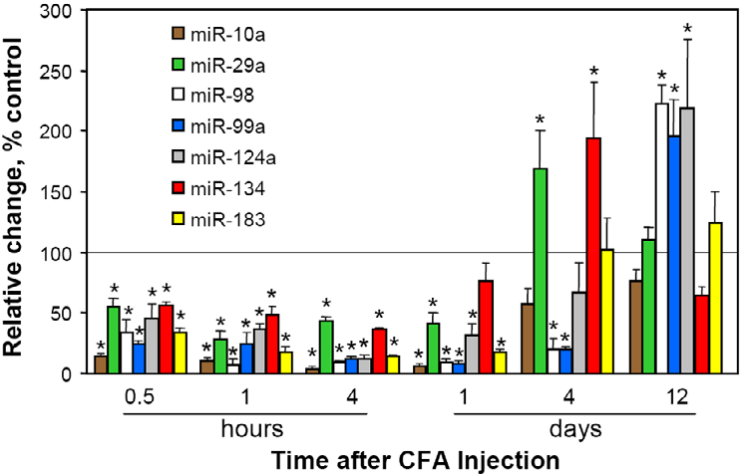
**Quantitative downregulation of mature miRNAs in the TG V3 following unilateral CFA injection**. Total RNA was extracted by the Absolutely RNA kit (Stratagene, Lo Jolla, CA) and 10 ng of RNA was used to generate cDNA using a TaqMan miRNA RT kit and TaqMan miRNA primers specific to mature miRNA (ABI). Each TaqMan PCR reaction contained cDNA derived from 0.88 ng RNA. Changes in miRNA level were calculated by a ΔΔCt method [4] and are presented as percentage of control (the basal level in naïve animal) in mean + s.e. from 6 to 12 samples. Each sample was measured in triplicate. Sample differences were examined by one-way ANOVA separately for each miRNA. *: P < 0.05 when compared to the control.

We then analyzed the level of detectable miRNA species in V3 of animals inflamed by CFA at various post-injection time points. To correct sample loading and RT efficiency, we normalized miRNA signal with the U6 RNA and the glyceraldehyde-3-phosphate dehydrogenase mRNA. These internal controls in our validation assays remained stable along the tested time period (data not shown). Both yielded similar results. But, since U6 RNA was detected by the same type of TaqMan assay as miRNAs, in Fig. 1 we show the results normalized by this small RNA. All tested miRNAs were significantly downregulated within 4 hr after CFA. The extent of downregulation can be categorized into three groups: one retaining less than 5% of the basal miRNA level in naïve animal (miR-10a, -98); one maintaining 5~15% (miR-99, -124a, -183) and one showing more than 25% left (miR-29a, -134). In the time course of downregulation, miR-10a, -98, -99, and -124a showed a long duration (~24 hr) of downregulation, while miR-134 nearly fully recovered by this time (P > 0.05 compared to the basal level). By day 12, all tested miRNAs were completely reversed to a level similar to or higher than the basal level. We noticed that miR-29a, -99, -124a, and -134 in inflamed animals reached a much higher level than that in naïve animals. This phenomenon was often seen during mRNA regulation [24] and the rebounded change will be brought back to the base level eventually, e.g. miR-29a and -134 in this study. In contrast, saline-injected animals did not show a significant change in tested miRNAs in the ipsilateral V3 division. The downregulation was also not seen in the contralateral V3 or the ipsilateral V1/V2 divisions of the TGs in the same CFA-injected animals (data not shown). More importantly, from the same batch of RNA samples, calcitonin gene-related peptide mRNA was shown to be upregulated 30 min after CFA, which correlates with the development of mechanical allodynia [4]. Therefore, we believe that miRNA downregulation is specifically associated with the CFA-induced inflammation and allodynia.

**Figure 2:**
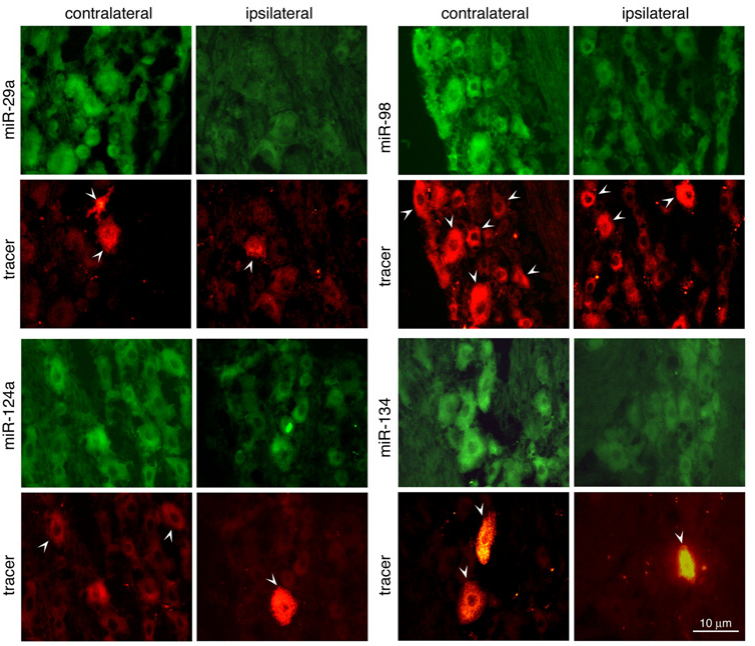
**Distribution and downregulation of miRNA in TG**. TG tissues were obtained from animals inflamed by CFA for 4 hr and *in situ *hybridization was performed with 5' biotin labeled LNA probes according to the protocol recommended by the manufacturer (Exiqon). Bound probes were detected by Cy3-streptavidin for green fluorescence while the tracer rhodamine-conjugated dextran produced red fluorescence. White arrowhead indicates tracer labeled cells.

Next, we addressed whether the neurons innervating the injected muscle express these miRNAs and, if so, whether these miRNAs respond to inflammation. We injected a retrograde neuronal tracer, rhodamine-conjugated dextran (Invitrogen, Carlsbad, CA), bilaterally into the masseter muscle 5 days before the CFA injection [19]. Four hrs after the CFA injection, we perfused the animals (n = 6) and examined miRNA in TGs using biotin-labeled locked nucleic acid (LNA) probes (Exiqon, Vedbaek, Denmark) in *in situ *hybridization. Neurons innervating the injected muscles exhibited rhodamine signal and probes hybridized to miRNA were visualized by Cy3-conjugated streptavidin (Fig. 2). Consistent with the real-time RT-PCR results, downregulation of tested miRNAs was found in TG neurons including rhodamine-positive cells that innervate the inflamed muscle. In addition, miRNA signals were associated with neurons of all sizes although large neurons seem to exhibit more signals. Interestingly, LNA probes for miR-143 and -153 again did not produce any positive signal (data not shown). These results together with the TaqMan assays suggest that these miRNA species are present at a very low level or not expressed in TG V3 neurons.

**Figure 3:**
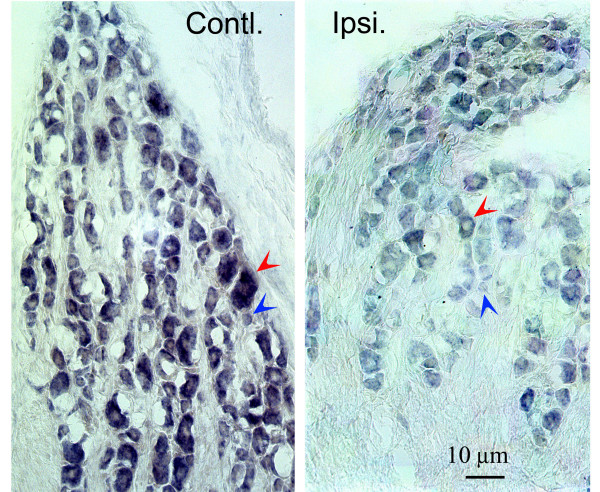
**miR-134 level in the ispi- and contralateral TG**. The LNA probe of miR-134 was tailed by digoxigenin-dUTP, and visualized by a detection kit (Roche). Ipsi = ipsilateral; Contl = contralateral. Red arrowhead: large neuron; blue arrowhead: small neuron.

To support the above observations, we tailed miR-134 and -143 LNA probes with digoxigenin-dUTP and viewed miRNA signal in *in situ *hybridization with a colorimetric method, which in general produces better cellular morphology. As shown in Fig. 3, these experiments show a downregulation of miRNA comparable to that obtained by the fluorescent method. The stability of precipitated color further confirmed the quantitative change in TG neurons. Glial cells and other nonneuronal cells in TG did not show detectable miR-134. Again, the miR-143 probe did not reveal any signal in these experiments (data not shown).

## Conclusion

The LNA probe is virtually antisense to the mature miRNA sequence that is present in both pre- and mature miRNA in the cytosol [7]. Therefore, the signal obtained from *in situ *hybridization represents both types of molecules of a specific miRNA. The results of the TaqMan assay and *in situ *hybridization suggest that the downregulation occurred either at both pre- and mature miRNA levels or only at the mature miRNA level if the latter is the major form in the TG.

The present study for the first time demonstrates miRNA expression in the peripheral nervous system at the mature miRNA level and with single cell resolution. Most importantly, we observed that several miRNA molecules, likely in the mature form, are regulated by an inflammatory irritant and their changes are correlated with the development of allodynia. Although the detailed mechanism underlying this regulation remains unknown at this stage, the RNA polymerase II (Pol II) is found to govern the transcription of the most miRNA genes [7], and inflammation is known to induce rapid expression or modification of several transcription factors such as c-fos and CREB in neurons [2,3,6]. These factors may negatively regulate Pol II activity in neurons under certain conditions.

Discovery of miRNA downregulation provides a novel view of the mechanism(s) underlying inflammatory pain. Downregulation of miRNA releases the translation inhibition of target mRNAs, thus yielding more proteins that may be relevant to the development and/or maintenance of inflammatory pain. However, these initial studies only demonstrated downregulation of a few selected miRNAs in TG sensory neurons during the time when allodynia occurred [4]. Whether this miRNA downregulation is mechanistically involved in inflammatory pain cannot be addressed by the present study. How miRNA participates in inflammatory pain relies, at least in part, on the elucidation of their target mRNAs and/or on the impact of manipulated levels of specific miRNA on nociception. The former is a complex question. Even though several programs have been developed to predict the potential targets for a given miRNA [23,25-27], systematic studies are needed to thoroughly address this question.

## Competing interests

The author(s) declare that they have no competing interests.

## Authors' contributions

GB is responsible for initiation, experimental design, performance of real-time RT-PCR assays, data analysis, and drafting and finalizing the manuscript of this project. RA contributed to experimental design, animal experiments and RNA extraction as well as manuscript editing. DW conducted *in situ *hybridization experiments and data analysis. DD contributed to support, statistical analysis, and manuscript editing. All authors have read and agreed with the final manuscript.
